# Assessment of the Stability and Nutritional Quality of Hemp Oil and Pumpkin Seed Oil Blends

**DOI:** 10.3390/foods13233813

**Published:** 2024-11-26

**Authors:** Marta Siol, Natalia Chołuj, Diana Mańko-Jurkowska, Joanna Bryś

**Affiliations:** 1 Department of Chemistry, Institute of Food Sciences, Warsaw University of Life Sciences, 02-776 Warsaw, Poland; marta_siol@sggw.edu.pl (M.S.); diana_manko-jurkowska@sggw.edu.pl (D.M.-J.); 2 Faculty of Food Technology, Warsaw University of Life Sciences, 02-776 Warsaw, Poland; natalia.choluj10@gmail.com

**Keywords:** hemp oil, pumpkin seed oil, fatty acid composition and distribution, hydrolytic properties, oxidative properties

## Abstract

This study characterized the quality of hemp oil (HO) and pumpkin seed oil (PO) and their blends before and after 2 and 4 months of storage at refrigerated and room temperature, without access to light and oxygen. The analyses included determining the acid value, peroxide value, fatty acid (FA) composition, and FA distribution in triacylglycerol (TAG) molecules. Pressure differential scanning calorimetry (PDSC) was used to assess the oxidative stability of oils and their blends. This study also evaluated the nutritional potential of hemp oil and pumpkin seed oil blends, as atherogenicity, thrombogenicity, and health-promoting indices and hypocholesterolaemic/hypercholesterolaemic ratio were calculated. The tested samples differed in properties depending on the storage time and temperature. The optimal choice was a blend of 50% hemp oil (HO) and 50% pumpkin oil (PO). This mixture demonstrated the desired fatty acid composition, satisfactory acid and peroxide values, and a relatively good oxidation induction time during storage. Despite the unfavorable distribution of FAs in TAG molecules, it was characterized by a balanced ratio of n-3 to n-6 acids. It was also concluded that research on HO and PO mixtures should be continued due to the potential synergistic effect of their bioactive substances.

## 1. Introduction

Hemp oil (HO), derived from the seeds of *Cannabis sativa* L., is renowned for its nutritional, health-promoting, and bioactive properties. Due to the psychoactive effects of Δ9-tetrahydrocannabinol (THC), present in the seeds of *C. sativa* L., the production of seeds and their derived products is regulated by European legislation. This legislation stipulates that hemp cultivation is permitted only if it contains less than 0.2% (*w*/*w*) THC (Regulation 1307/2013) [1]. Depending on the variety and cultivation conditions, hemp seeds contain approximately 28–35% oil [2]. Unlike other vegetable oils, hemp oil is particularly rich in essential fatty acids (EFAs), omega-6 and omega-3. The n-3 and n-6 fatty acids (FAs) are present in a favorable ratio of 1:3. This characteristic imparts several beneficial effects on the human body, such as anti-inflammatory, anti-cancer, antithrombotic properties, and regulation of metabolic activity [3]. Over 90% of the fatty acid composition of hemp oil consists of unsaturated FAs [4,5]. Hemp oil is especially prone to oxidation due to its high content of polyunsaturated fatty acids (PUFAs) and the presence of a significant amount of chlorophyll pigments (9 mg/100 g). The high chlorophyll content contributes to its green color [6]. Chlorophyll and its derivatives originate from the plant seeds and are not removed during the cold pressing. They are susceptible to photooxidation and exert prooxidative effects, reducing the oxidative stability of the oil and accelerating rancidity, thereby shortening its shelf life [6,7]. As a result of these pigments, under the influence of light, HO may change color from green to yellow [8]. Additionally, HO contains antioxidants such as carotenoids, tocopherols, phenolic compounds, and phytosterols. All these components mitigate the action of free radicals responsible for cell membrane damage and skin-aging processes [5]. The presence of carotenoids—mainly β-carotene (3.36–5.34 mg/100 g)—may contribute to the protection of chlorophyll pigments from photooxidation, thereby preventing color change during oil storage [8,9]. The oxidative stability of hemp oil is favorable, and it is primarily influenced by the ratio of linoleic to oleic acid, naturally occurring bioactive components, and the content of chlorophyll pigments [10,11].

Pumpkin seed oil (PO) is characterized by its high nutritional value, attributable to its favorable fatty acid composition and diversity of bioactive compounds [12]. It supplements the diet with EFAs, vitamins, phytosterols, chlorophyll, and trace elements such as zinc and selenium [13]. The oil is rich in mono- and polyunsaturated fatty acids (approximately 80% of all FAs), with linoleic acid (31–66%) and oleic acid (15–49%) being predominant. PO contains high phytosterols, predominantly β-sitosterol (84.40–116.33 mg/100 g). It is an excellent source of carotenoids (lutein, zeaxanthin—totaling 0.25–0.66 mg/100 g of oil), tocopherols (mainly δ-tocopherol—approx. 177 mg/100 g of oil), and phenolic compounds (primarily syringic acid—approx. 7.96 mg/100 g of oil—and ferulic acid—approx. 4.99 mg/100 g of oil) [10,14,15,16]. This oil also contains squalene (approx. 600 mg/100 g), a precursor of all steroids in plant and animal cells, significantly influencing its oxidative stability [11]. PO provides numerous health benefits, including aiding in the management of diabetes, hypertension, and cancer, as well as exhibiting antioxidant, anti-inflammatory, and antibacterial properties. Its antioxidant properties may improve fertility, prevent atherosclerosis, and stimulate fat metabolism [13,14,17]. Among its remarkable benefits is the prevention of prostate and urinary tract diseases [18]. The oil has a dark green-red color [19], resulting from the presence of carotenoids and chlorophylls [10,20].

Due to changing tastes and increasing consumer demands, the food industry seeks new technological solutions to produce lipids with improved nutritional and functional properties. Blending edible oils is one of the simplest methods for creating new, specific product formulations in the food industry [21]. Through the combination of the characteristics of two oils into one, it is possible to produce edible oils with guaranteed quality and beneficial properties [22]. This is an economical way to improve their oxidative stability and organoleptic and physicochemical properties [23]. Understanding the properties of oils and selecting them appropriately for blending can limit hydrolysis, oxidation, thermal decomposition, cyclization, and isomerization processes, as well as increase the smoke point and reduce their viscosity. Blending oils changes and improves the fatty acid composition, thus eliminating the need for hydrogenation of FAs and the formation of trans-fatty acids [24]. Oils with relatively good oxidation resistance are mixed with oils that have weaker resistance. As a result, the final product is enriched with bioactive compounds and gains greater oxidative stability. The literature lacks data regarding the nutritional properties and health benefits of non-traditional vegetable oils blends that would provide a favorable fatty acid composition (richness in PUFA and EFA) while maintaining oxidative and hydrolytic stability. In recent years, extensive phytochemical, mechanistic, and clinical studies have been conducted on PO and HO, and more and more claims and statements are being made to support these oils as functional foods.

This study aimed to accurately evaluate the quality of commercial hemp and pumpkin seed oils and their blends before and after 2 and 4 months of typical household storage conditions. Parameters such as the composition and distribution of fatty acids in the structure of triacylglycerols and the oxidative and hydrolytic stability were considered. Moreover, health indices (atherogenicity, thrombogenicity, health-promoting indices, and hypocholesterolaemic/hypercholesterolaemic ratio) were evaluated.

## 2. Materials and Methods

### 2.1. Materials

Two commercial oils (hemp and pumpkin seed) and their three mixtures prepared in the 3:1, 1:1, and 1:3 (*v*/*v*) proportions of HO to PO were tested in this study. The oils were purchased from the same producer within their shelf life and tested before expiration. According to manufacturers’ declarations, the oils were cold-pressed, non-refined, not-filtrated, and were 100% natural without additional antioxidants. The oils were produced from raw materials grown in Poland. For cultivation, the certified hull-less seeded Styrian (Australian) oil-pumpkin variety—Gleisdorfer Ölkürbis (*C. pepo*)—and seeds of Finola cultivar of hemp (*Cannabis sativa* L.) were used. According to the manufacturer’s declaration and applicable law, the THC content in the hemp oil was less than 7.5 mg/kg [25]. Both oils were supplied in dark glass bottles. The oils were stored for 4 months at 4 ± 1 °C (according to the manufacturer’s recommendations) and at 20 ± 2 °C (room temperature). The first determinations were made immediately after purchasing commercial oils. The next ones were made while storing the oils and their blends without access to light and oxygen after 2 and 4 months.

### 2.2. Methods

#### 2.2.1. Determination of Fatty Acid Composition by Gas Chromatography

The composition of fatty acids (the abundance percentage of each FA) present in oils was carried out according to the ISO method [26]. The method described by Bryś et al. [27] was used to determine the fatty acid profile of the tested samples. A YL6100 GC Clarity gas chromatograph (Young Lin Bldg., Anyang, Republic of Korea) was used, which was equipped with a GC BPX70 capillary column filled with a stationary phase, 0.6 × 10^2^ m long, with an internal diameter of 0.25 × 10^−3^ m and film thickness 0.25 × 10^−6^ m.

#### 2.2.2. Health Indices of Oils

The FA composition was used to calculate the health indices of the tested oils. The atherogenicity index (AI) and thrombogenicity index (TI) were obtained from Equations (1) and (2), respectively [28], and the health-promoting index (HPI) and hypocholesterolaemic/hypercholesterolaemic ratio (h/H) were obtained from Equations (3) and (4) [29,30]:

(1) $$AI=\frac{C12:0+\left(4\times C14:0\right)+C16:0}{\sum UFA}$$

(2) $$TI=\frac{C14:0+C16:0+C18:0}{0.5\times \sum MUFA+\left(0.5\times \sum n-6PUFA\right)+\left(3\times \sum n-3PUFA\right)+\frac{n-3}{n-6}}$$

(3) $$HPI=\frac{\sum UFA}{C12:0+\left(4\times C14:0\right)+C16:0}$$

(4) $$h/H=\frac{cis-C18:1+\sum PUFA}{C12:0+C14:0+C16:0}$$

#### 2.2.3. Distribution of Fatty Acids in Triacylglycerols Using Enzymatic Hydrolysis

The enzymatic hydrolysis method described by Brzezińska et al. [31] was used to determine the structure of triacylglycerols of the tested samples. Based on the compositions of the isolated *sn*-2 monoacylglycerols (MAGs) and the starting TAGs, the composition of the FAs in the *sn*-1,3 positions was determined. The following Equations were used:

(5) $$sn-2=\frac{({FA}_{in\;sn-2\;MAG})\times 100\%}{3\times \left({FA}_{in\;TAG}\right)}$$

(6) $$sn-1,3=\frac{3\times ({FA}_{in\;TAG})-({FA}_{in\;sn-\;2MAG})}{2}$$

where:

*sn*-1,3—the content of a given FA in *sn*-1 and *sn*-3 positions [%];

FA_in TAG_—the content of a given FA in the starting triacylglycerols [%];

FA_in *sn*-2 MAG_—the content of a given FA in *sn*-2 monoacylglycerols [%].

The determinations were made immediately after purchasing the commercial oils.

#### 2.2.4. Determination of Acid and Peroxide Values by Potentiometric Titration Method

The degree of hydrolysis of the investigated oils was determined by the acid value (AV) according to the AOCS method (AOCS Official Method Te 1a-64) [32]. The content of primary oxidation products of the oils was examined via the peroxide content according to the AOCS method (AOCS Cd 8b-90) [33] using an automatic titrator, TitraLab AT1000 Series (HACH LANGE, Wroclaw, Poland).

#### 2.2.5. Determination of Oxidative Stability Using Pressure Differential Scanning Calorimetry

Pressure differential scanning calorimetry using PDSC Q20 (DSC Q20 TA Instruments, New Castel, DE, USA) was used to determine the maximum oxidation time (τ_max_) for the tested oils. Weighed oil samples (3–4 mg) were placed on an open aluminum pan in the heating sample chamber of the PDSC cell. Experiments were performed under an oxygen atmosphere at an initial pressure of around 1400 kPa [34]. The isothermal temperature for each sample was programmed at 120 °C. Obtained PDSC curves were analyzed using TA Instruments Universal Analysis 2000 software. The PDSC oxidation time was determined based on the maximum rate of heat flow with an accuracy of 0.005.

### 2.3. Statistical Analysis

The results measurements were repeated three times and presented as mean ± SD. Statistical analyses were performed using the program STATISTICA 13.3 (StatSoft, Krakow, Poland), using an analysis of variance (ANOVA) and Tukey test at a significance level of α = 0.05. A Pearson’s linear correlation analysis was also used to determine the relationship between selected parameters.

## 3. Results and Discussion

### 3.1. Fatty Acid Profile

The composition of fatty acids is important in determining oils’ nutritional value, technological properties, and oxidative stability [21,35]. A higher content of saturated fatty acids (SFAs) is associated with better oxidative stability of fat; however, from a nutritional point of view, it is advisable to limit the amount of SFAs supplied to the body and replace them with unsaturated ones, especially essential fatty acids. EFAs can help in the fight against cardiovascular diseases, obesity, type II diabetes, cancer, and even depression [36,37].

**Figure 1:**
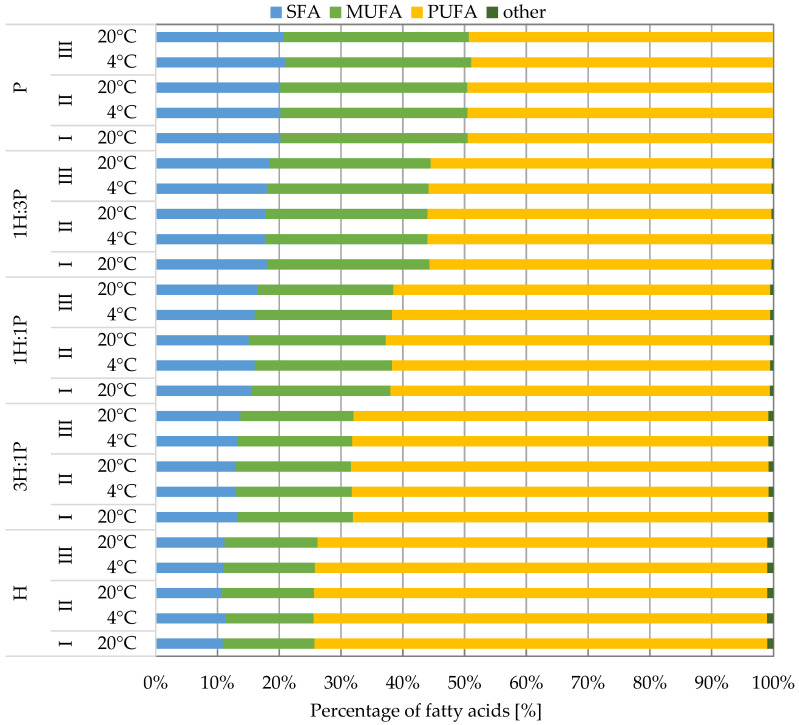
The content of saturated (SFAS), monounsaturated (MUFAS), and polyunsaturated fatty acids (PUFAS) for tested oils and their mixtures, where H—hemp oil, 3H:1P—3:1 (*v*/*v*) hemp oil to pumpkin seed oil mixture, 1H:1P—1:1 (*v*/*v*) hemp oil to pumpkin seed oil mixture, 1H:3P—1:3 (*v*/*v*) hemp oil to pumpkin seed oil mixture, P—pumpkin seed oil immediately after opening (I) and after two (II) and four months (III) of storage at 4 °C and 20 °C.

Figure 1 shows the average percentage of SFA in the tested oils and their mixtures. Before storage, HO had the lowest content of SFA (10.89 ± 0.56%), and PO had the highest content (20.21 ± 0.24%). As the proportion of pumpkin in the mixture increased, the percentage of SFA increased. In the research of Petrović et al. [4], the percentage of SFA in several hemp oil samples was 9.43–11.33%. Their content in pumpkin seed oil in the study by Ciećko et al. [38] was 18.25% and in Bielecka et al. [39] was 20.00%.

Typically, there were no statistically significant differences between the average SFA content before and after subjecting the oils and their mixtures to storage tests under various temperature conditions (*p* > 0.05). The exception was the 1H:1P mixture, in which SFA decreased in the second testing period at a temperature of 20 °C.

Before storage, the hemp oil had the lowest content of monosaturated fatty acids (MUFAs) (14.83 ± 0.22%), and pumpkin seed oil had the highest content (30.33 ± 0.04%). The difference between these values, as in the case of SFA, was twofold. The higher the content of PO in the mixture, the higher the content of MUFA in it. The percentage of MUFA in hemp oil was very similar to the result obtained by Petrović et al. [4] (10.29–14.57%). In contrast, in pumpkin seed oil, their content in the study by Ciećko et al. [38] was 38.43%, and in Bielecka et al. [39], it was 25.9%. It should be emphasized that when comparing different pumpkin cultivars, the highest MUFAs (and the highest oleic acid content) were found in Gleisdorfer Ölkürbis, which is especially useful for the production of high-quality pumpkin oil.

Compared to the MUFAs, the hemp oil had the highest content of polyunsaturated fatty acids, PUFAs (73.30 ± 0.37%), and the pumpkin seed oil had the lowest content (49.44 ± 0.20%) before storage. The greater the addition of PO to the mixture, the lower the percentage of PUFA was. It should be mentioned that the literature data indicate that the ‘Finola’ variety had a significantly lower PUFA content than oils obtained from seeds of other cultivars of Cannabis sativa. Research by Petrović et al. [4] showed that PUFA accounted for 80% of the tested hemp oils. Their content in pumpkin seed oil was 43.26% in the study by Ciećko et al. [38], and in Bielecka et al. [39], it was 54.1%. Any observed differences in the FA content may be attributed to the use of oil from different varieties of hemp and pumpkin in the studies.

It should also be noted that the PUFA/SFA ratio (also known as polyene index) can be treated as a measure of the extent of polyunsaturation of oils/blends and their tendency to undergo autoxidation. Meanwhile, PUFA/SFA changed in the following order: H > 3H:1P > 1H:1P > 1H:3P > P (6.74 > 5.07 > 3.96 > 3.07 > 2.45, respectively). It indicated that the more pumpkin oil in the mixture, the less susceptible it is to autoxidation.

The dominant fatty acids in HO were linoleic acid (~52%), α-linolenic acid (~17%), oleic acid (~14%), palmitic acid (~6%), and stearic acid (~4%). In most previous studies, very similar or the same results were obtained, i.e., 52.13–57.26% (linoleic), 14.79–19.91% (α-linolenic), 8.42–13.44% (oleic), 5.91–7.38% (palmitic), and 2.13–3.42% (stearic) [4,36]. In turn, the FA composition of PO was dominated by linoleic acid (~49%), oleic acid (~30%), palmitic acid (~12%), and stearic acid (~8%). Similar results were obtained by Tańska et al. [15], who analyzed oil from hull-less seed pumpkins (50.9–62.9%, 21.4–32.6%, 9.9–11.9%, and 4.1–5.8%, respectively) and Ciećko et al. [38] examining pumpkin seed oil (42.98%, 37.55%, 11.75%, and 5.71%, respectively).

Statistical analysis revealed no significant influence of time and temperature on the changes in the content of individual types of FAs during the storage of oils and their mixtures. The higher the proportion of PO in the sample, the lower the percentage of PUFA and the greater the proportion of SFA and MUFA.

**Figure 2:**
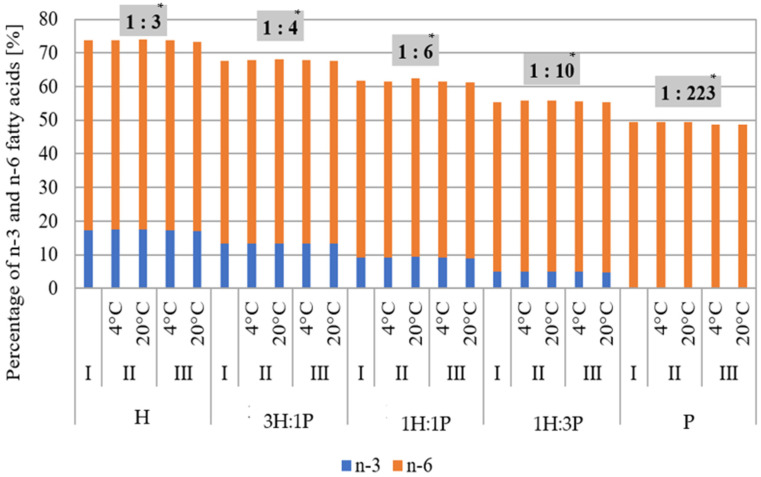
The content of n-3 and n-6 fatty acids for tested oils and their mixtures, where H—hemp oil, 3H:1P—3:1 (*v*/*v*) hemp oil to pumpkin seed oil mixture, 1H:1P—1:1 (*v*/*v*) hemp oil to pumpkin seed oil mixture, 1H:3P—1:3 (*v*/*v*) hemp oil to pumpkin seed oil mixture, P—pumpkin seed oil immediately after opening (I) and after two (II) and four months (III) of storage at 4 °C and 20 °C. * Ratio of n-3 to n-6 fatty acids.

Figure 2 shows the average percentage of n-3 and n-6 FAs and their ratio in the tested oils and mixtures.

As the content of PO in the tested samples increased, the percentage of omega-3 and omega-6 acids decreased, and their proportion also decreased. The HO was characterized by a well-balanced ratio of n-3 and n-6 acids (1:3), also confirmed by the literature data [4,40,41]. Furthermore, the recommended ratios of these acids were observed in mixtures 3H:1P (1:4) and 1H:1P (1:6). This indicates the favorable nutritional properties of these mixtures—their regular consumption could provide health benefits to consumers.

Based on the FA profile (the results are shown in Tables S1 and S2 in Supplementary Files), it is possible to calculate dietary indices for the risk of developing cardiovascular disease, such as the index of atherogenicity (AI) and the index of thrombogenicity (TI). The oils recommended for consumption should present low atherogenicity (AI < 1.0) and thrombogenicity (TI < 0.5) indices and high health-promoting (HPI) and hypocholesterolaemic/hypercholesterolaemic ratio (h/H).

Considering the results obtained for the studied oils (Table 1), it can be stated that they are of favorable nutritional and health value, especially hemp oil. Moreover, the results showed that even the four-month storage period did not significantly change the values of the health indicators of the tested oils.

The obtained AI values for PO were twice as high as for HO and comparable to the AI obtained by Ulbricht and Southgate for olive oil (0.14) [42] and by Ying et al. for borage oil (0.18) [43]. In turn, the AI of hemp oil was comparable to the data obtained by Górska et al. [44] for strawberry and cranberry seed oils (0.057 and 0.065, respectively). Consumption of products with low AI is correlated with a reduction in total cholesterol and low-density lipoprotein cholesterol in human blood plasma [30,45,46].

The TI value of HO was almost five times lower than TI for PO and close to the TI indices obtained by Ratusz et al. [47] for cold-pressed camelina oils. Hemp oil was also characterized by the highest HPI and h/H indices (15.35 and 15.55, respectively). Also, Ratusz et al. for camelian oil [47] and Siol et al. [45] for pomegranate cold-pressed oils obtained comparable h/H values. Consuming products with a low TI and high h/H may be beneficial in preventing cardiovascular heart disease [30,45,46].

### 3.2. Fatty Acid Distribution

The digestion of fats involves primarily the enzymatic hydrolysis of TAGs found in the diet. This digestion occurs mainly in the duodenum, and the action of pancreatic and lipoprotein lipases is primarily directed at fatty acids in the *sn*-1 and *sn*-3 positions of TAGs, which results in the formation of free fatty acids and *sn*-2 MAGs after hydrolysis. The type of fatty acid and its location in TAG largely determine the physical behavior of fats from our diet but also affect their absorption. Short- and medium-chain FAs originating from the *sn*-1 and *sn*-3 positions and long-chain FAs containing more than 12 carbons are subject to different absorption pathways, similar to *sn*-2 MAGs. The absorption and metabolism of long-chain FAs released from the *sn*-1 and *sn*-3 positions are much slower and require proteins, whereas *sn*-2 MAGs are absorbed by passive diffusion. Additionally, long-chain FAs, especially saturated ones, have low absorption rates because their melting point is higher than body temperature, and they can form insoluble calcium soaps. However, short- (C4–C6) and medium-chain (C8–C10) FAs dissolve in intestinal fluids and are absorbed directly into the portal system. Once in the portal circulation, these acids form complexes with albumin and are transported to the liver for oxidation. Therefore, fat digestibility largely depends on the structure of TAGs [48,49].

Methods that utilize enzyme regiospecificity and stereospecificity are employed to determine the structure of triacylglycerols. One of the most frequently used biocatalysts for this purpose is pancreatic lipase, which is also involved in the digestion of fats in the human digestive tract. It is capable of deacylation of TAG only in external positions and does not distinguish between them. Therefore, it is assumed that they are equivalent (the share of FA in the middle position of over 33.3% means that it prefers the internal TAG positions) [50].

**Figure 3:**
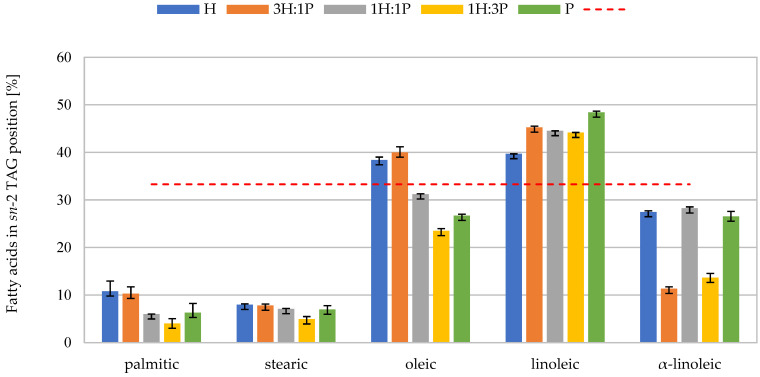
The percentage of a given fatty acid in *sn*-2 position of triacylglycerols (TAGs) of tested oils and their mixtures, where H—hemp oil, 3H:1P—3:1 (*v*/*v*) hemp oil to pumpkin seed oil mixture, 1H:1P—1:1 (*v*/*v*) hemp oil to pumpkin seed oil mixture, 1H:3P—1:3 (*v*/*v*) hemp oil to pumpkin seed oil mixture, P—pumpkin seed oil. The dashed line indicates the statistical (even) distribution of FA between three TAGs positions (33%).

An improper structure of TAGs in oils can lead to reduced fat absorption, loss of EFAs, and excessive excretion of calcium and other salts from the body. The most favorable situation occurs when in position in the middle, there are EFAs, and in the external positions, there are short- and medium-chain FAs [50,51]. The analysis included the dominant FAs found in the tested oils, i.e., palmitic acid (C16:0), stearic acid (C18:0), oleic acid (C18:1), linoleic acid (C18:2), and α-linolenic acid (C18:3 n-3, ALA). They were all long-chain FAs. Figure 3 displays the average percentage of dominant FAs in the *sn*-2 position in TAGs in the tested oils and their mixtures.

Linoleic acid was the most abundant acid in the internal position of TAGs across all tested samples. Its highest share in the *sn*-2 position was recorded in PO (48.42 ± 0.26%) and the lowest in HO (39.67 ± 0.03%). Oleic acid predominantly occupied the middle positions of TAGs in HO (38.41 ± 0.62%) and the 3H:1P mixture (39.99 ± 1.19%). In external positions, its share increased successively: in the 1H:3P mixture (23.52 ± 0.46%), in PO (26.69 ± 0.30%), and in the 1H:1P mixture (31.19 ± 0.11%). α-linolenic acid showed a preference for external TAG positions in all tested oils and their mixtures. There were no statistically significant differences between its share in the internal position of TAGs in HO, in the 1H:1P mixture, and in PO (approx. 27%) (*p* > 0.05). In the 3H:1P and 1H:3P mixtures, the share of ALA was more than twice as low (approx. 12%).

Saturated acids predominantly occurred mainly in the external positions of TAGs. The share of palmitic acid in the middle TAG position was the lowest in the 1H:3P mixture (4.03 ± 1.00%) and the highest in the HO and the 3H:1P mixture (~10.50%). In turn, the share of stearic acid in the internal TAG position was significantly the same in all samples (approx. 7%) except for the 1H:3P mixture (4.94 ± 0.55%).

Tringaniello et al. [52], in their examination of hemp oil samples, determined the content of individual FAs in the internal position of TAGs at the following levels: approx. 68% (linoleic), 15% (oleic) and 15% (α-linolenic). The TAG structure identified by the authors was partially similar to that found in the analyzed HO: according to their results, the C18:2 acid was placed in the *sn*-2 position of the TAG, and the C16:0 acid was located mainly in the *sn*-1,3. Yao et al. [53] determined the composition of individual FAs in the *sn*-2 TAG position in pumpkin seed oil at the following levels: 51% (linoleic), 24% (oleic) and 6% (palmitic). Consistent with our findings, these authors indicated that linoleic acid prefers the internal TAG position, while oleic and palmitic acids are more likely to occupy the external positions.

From the nutritional point of view, the distribution of FAs between TAG molecules in the tested oils and their mixtures was not favorable. Saturated FAs were found exclusively in the external positions across all samples. In the 1H:1P and 1H:3P mixtures, as well as in PO, oleic acid also preferred the external positions. Therefore, after consuming the mentioned oils or their mixtures, these SFAs placed in the *sn*-1 and *sn*-3 positions would be lost along with the feces (if after hydrolysis they form insoluble salts with Ca^2+^ ions).

### 3.3. Evaluation of Oil Quality

Cold-pressed oils rich in PUFA and vegetable oil blends tend to undergo rapid oxidation during storage, affected by external (e.g., temperature, light exposure, oxygen availability) and internal factors (e.g., the variety, the cultivation area/geographical origin of raw material) [54]. The oxidation of oils has important effects on their nutritional properties and quality.

Cold-pressed oils in households are commonly storage at room temperature or according manufacture’s recommendations—under refrigeration [55]. It is known that the type of bottles (material, color) in which oils are packaged also affects the quality of the oil during storage. According to Robertson et al. [56] glass containers are able to completely prevent O_2_ permeation. Dark glass bottles (dark green, brown, amber) are recommended in the food, cosmetics, and medicines industry to protect light-sensitive ingredients [57]. Dark glass absorbs most UV radiation, eliminating its destructive effects. Wroniak et al. [55] indicated a similar loss in quality in the dark and the presence of light at room temperature in containers made of amber glass and amber PET. In the present study, oils were stored in original, dark glass bottles for 4 months at average temperatures of about 4 °C (refrigeration conditions) and 20 °C (room temperature) and tested regarding their peroxide value, acid value, and oxidative stability.

#### 3.3.1. The Acid Value

Hydrolysis of TAGs results in the formation of diacylglycerols (DAGs), monoacylglycerols, and free fatty acids (FFAs), which are more susceptible to autoxidation than esterified fatty acids. Their ability to accelerate oxidation results from reducing the surface tension of oils and increasing the rate of oxygen diffusion from the free space above the oil to the oil [58,59]. The favorable hydrolytic properties of oils are determined by the good quality of the seeds, their storage conditions, and the composition of FAs [59].

The acid value (AV) indicates the freshness of fats, describing the degree of their hydrolysis. It is also a measure of the content of FFAs. According to Codex Alimentarius, the AV of cold-pressed vegetable oils should not exceed 4 mg KOH/g fat [60]. The average values of the AV of the tested oils and their mixtures are presented in Table 2.

Before storage, PO had the lowest AV (0.58 ± 0.01 mg KOH/g fat), and HO had the highest (4.25 ± 0.01). Strong correlations were observed between the AV and the composition of FAs: negative for SFA (r = −0.96) and MUFA (r = −0.98) and positive for PUFA (r = 0.97), which means that as the content of SFA and MUFA increased and the content of PUFA decreased in individual samples, their AVs decreased. Therefore, the lower degree of hydrolysis of PO compared to HO and its gradual decrease with the increase in the content of the PO in the mixtures could be related to the FA profile of the analyzed oils.

Hemp oil was characterized by a very high content of PUFAs, whereas PO contained relatively high levels of SFAs. All samples, except HO, had the AV lower than 4 mg KOH/g fat. The value of the acid number of HO above the permissible value for cold-pressed oils was probably due to the poor quality of hemp seeds, which may have been of poorer quality already at the time of harvest and/or been stored improperly (at too low or too high a temperature) and for too long. These factors may have contributed to the damage of the seeds and their absorption of more water, which, in combination with the native seed enzymes and lipolytic microorganisms, could have led to the breakdown of TAGs into MAGs, DAGs, and, most importantly, from the point of view of the acid value, into FFAs [5].

According to Oleksy et al. [61], the AV for hemp oil was 5.4 ± 0.16 mg KOH/g fat, which is higher than in the case of the samples studied in this study. This discrepancy could be attributed to a different variety of hemp seeds used to pressing the oil or their poorer quality. In contrast, Olesińska et al. [5], in their research on the properties of hemp oil obtained using various methods and from various sources, determined the AV at the level of 1.2 mg KOH/g fat (oil from retail sale) and 4.3 mg KOH/g fat (oil from a local manufacturer). The latter value aligns with the results obtained in this study. The commercial oil, which was the subject of research by Olesińska et al. [5], unlike oil intended for the local market, was characterized by a lower AV, not exceeding that permitted by Codex Alimentarius [60]. It should be underlined that more stringent requirements apply to commercial oils than oils for the local market.

The PO was produced from roasted seeds according to the manufacturer’s statement. Lipase activity and water content, one of the factors determining the degree of hydrolysis of oils, are reduced due to roasting or other thermal treatment. Consequently, the AV obtained for roasted pumpkin seed oil could be relatively low [21]. Lozada et al. [62] determined the AV of oil from Brazilian pumpkin seeds (previously dried in the sun) at the level of 0.15 ± 0.01 mg KOH /g fat, which is lower than that obtained in the present paper, so the seeds had to be of very good quality. It is possible that the manufacturer of the oil used in the study applied insufficient roasting conditions to inhibit the hydrolysis process. Kondratowicz-Pietruszka and Ostasz [63] determined the AV for oil from unroasted pumpkin seeds at the level of 2.00 ± 0.05 mg KOH/g of fat, and Singh and Kumar [64] at the level of 1.08 ± 0.01 mg KOH/g of fat, which confirms the positive effect of thermal treatment on the acid number of pumpkin seed oil.

After two months of storage, the AV of tested oils and their mixtures increased, and this increase was much greater in the case of oils stored at room temperature. In the case of PO, statistical analysis showed differences in AVs before and after two months of storage, although they were small. Moreover, there were no statistically significant differences between the average AV of oil stored at refrigerated and stored at room temperature (*p* > 0.05). Only in HO did the acid exceed the limit set by the Codex Alimentarius [60].

After four months of storage, the AV of all tested samples was higher than after two months of storage. Similarly to the studies on oils and mixtures stored for two months, the increase in AV for these samples stored at room temperature was usually greater than for those stored at refrigerated temperature. Comparing a sample of PO stored at refrigerated temperature to a sample stored at room temperature, it can be concluded that both after the first and second storage periods, there were no statistically significant differences between them in terms of the average AV (*p* > 0.05). Additionally, the differences in AVs for PO after 2 and 4 months of storage were statistically significant but nevertheless small. In HO and the 3H:1P mixture, the AV allowed by the Codex Alimentarius [60] was exceeded.

The acid value of the tested oils and their mixtures may have changed during storage due to the activity of lipolytic enzymes, which led to the hydrolysis of TAGs and the formation of more FFAs [21]. Hydrolysis is an endothermic reaction. Therefore, its intensity increases with increasing temperature, and water (trace amounts from seeds) mixes more easily with oils at higher temperatures, accelerating the degree of hydrolysis [65]. Prescha et al. [66] found no significant changes in the AV in hemp oil during its storage for 3 and 6 months at 20 °C (approx. 0.24 ± 0.13 mg KOH/g fat). The oil used was fresh (4 weeks from production), and the low degree of hydrolysis could be due to the very good quality of the seeds [66]. In Wroniak and Cenkier’s study [67], the AV of oil from roasted pumpkin seeds after 30 and 60 days of storage in refrigeration did not change significantly compared to the initial AV (approximately 2.2 mg KOH/g fat). This high AV could result from the low quality of the seeds and the use of a different pumpkin variety. In contrast, Nosenko et al. [68] reported that the AV of oil from unroasted pumpkin seeds remained stable for the first 40 days (approximately 0.9 mg KOH/g fat) and gradually increased over the next 56 days, reaching about 5 mg KOH/g fat. While the storage conditions were unspecified, it can be inferred that the temperature was higher and the seeds likely of lower quality. Additionally, the seeds were not subjected to heat treatment, so the water content and the activity of lipolytic enzymes were not reduced. In the study by Petkova et al. [69], lower (10 °C) and higher (25 °C) temperatures were used to store pumpkin seed oil (for 4 months). Their results confirmed the lack of influence of temperature and storage time on the value of AV (8.1–8.5 mg KOH/g fat), and the high values obtained were probably due to the very poor quality of the seeds and/or the use of oil for which a long time has passed since the extraction.

The hydrolysis degree increased as the HO content in the sample increased. The statistical analysis showed that using a higher temperature and extending the storage time increased the acid value. The exception was PO—the test parameter’s value was influenced only by storage time.

#### 3.3.2. The Peroxide Value

Fats oxidize under the influence of oxygen, high temperature, and light, and transition metals (iron, copper) can be catalysts for this process. Changes occur in all fats, but the most susceptible are lipids that contain many PUFAs. As a result, long-term storage of these products in conditions favorable to oxidation reduces their nutritional value and deteriorates their organoleptic properties. In addition to the FA profile, the susceptibility to oxidation is determined by the structure of TAGs and the presence of pro-oxidants (metal ions, chlorophyll derivatives, oxidation, and hydrolysis products) and antioxidants (e.g., tocopherols, carotenoids, squalene, phenolic compounds, phytosterols). The oxidation process produces primary oxidation products, which decompose into secondary oxidation products (including aldehydes and ketones), precursors of undesirable taste characteristics [23,39]. Lipid oxidation products are toxic to the human body, i.e., they cause oxidative stress, which initiates inflammation and influences the development of cardiovascular diseases, and are mutagenic and carcinogenic [70].

The peroxide value (PV) indicates the content of primary fat oxidation products (hydroperoxides), indicating the degree of oxidative changes. According to Codex Alimentarius, the PV of cold-pressed vegetable oils should not exceed 15 mEq O_2_/kg fat [60]. The average values of the PV of the tested oils and their mixtures are presented in Table 2.

Before storing the oils and their mixtures, the lowest PV was recorded for PO (4.81 ± 0.12 mEq O_2_/kg fat) and the highest for HO (7.24 ± 0.14). the statistical analysis showed a weak correlation between the PV and the FA profile in the tested samples (r = −0.33 (MUFA), r = −0.30 (SFA), r = 0.32 (PUFA)). Therefore, the lower PV of samples with a higher PO content may result from the high content of SFA and MUFA.

The literature data indicate that primary oxidation products are formed in larger amounts and in a shorter time in oils with a higher degree of unsaturation. The autoxidation rate depends on the alkyl radical formation rate, which is correlated with the composition of FAs [59]. Moreover, scientists report that hemp oil has a much higher amount of pro-oxidants in the form of chlorophyll pigments (9 mg/100 g) than pumpkin seed oil (less than 2 mg/kg of oil) [6,10]. In the tested oils and their mixtures, the photosensitized oxidation process and the influence of photosensitive dyes (chlorophylls) were minimized as much as possible because the samples were stored in a way that limited access to light. They could only be exposed to it for a short time during the analytical procedure. Therefore, the increase in peroxide content during storage was mainly caused by autoxidation [58,67]. Statistical analysis also showed a moderate positive correlation between the tested samples’ PV and AV (r = 0.47). Lipolysis accelerates fat oxidation because FFAs are more easily oxidized than FAs esterified with a glycerol molecule. Therefore, there is a synergism between oxidation and hydrolysis in the oil spoilage process. Additionally, oxidation is accelerated by lipolytic enzymes and water (derived from oilseeds). Removing water helps minimize the lipolysis process but does not completely inhibit oxidation, as it can still occur through an autocatalytic chemical mechanism [71]. Before storage, the tested mixtures were characterized by a lower PV than the maximum value specified by Codex Alimentarius [60].

Izzo et al. [8], examining the chemical composition of several hemp seed oils, determined their PV at 1.8–8.8 mEq O_2_/kg fat. The results confirmed significant differences in PV depending on the cannabis variety. As determined by the researchers, these results were comparable to those obtained in the present study. In the study by Olesińska et al. [5], the PV for hemp oil was approx. 4 mEq O_2_/kg fat (for oil from retail sales) and approx. 10 mEq O_2_/kg fat (for oil from a local producer). As in the case of the AV, the lower value of the PV of oil from the retail market was probably due to the more stringent requirements of its production and the possibility of using in tests oil stored for a shorter time after pressing compared to oil intended for the local market. Kondratowicz-Pietruszka and Ostasz [63], as well as Singh and Kumar [64], determined the PV of pumpkin oil (the seeds were not roasted) at a level similar to that obtained in this study—approx. 2.6 mEq O_2_/kg fat and 2.02 ± 0.15 mEq O_2_/kg fat, respectively. In the study by Wroniak and Cenkier [67], the PV of oil from roasted pumpkin seeds was 11.92 mEq O_2_/kg fat—almost three times higher than that obtained in our studies. The seeds were probably of poor quality and/or oils stored for a long time after pressing. In turn, Lyimo et al. [72] obtained PV very similar to that determined in this study (4.6 ± 0.01 mEq O_2_/kg fat). On the other hand, in the study by Gaca et al. [41], in the case of cold-pressed oils obtained from hemp and pumpkin seeds, the PV exceeded the standards contained in the Codex Alimentarius already before storage and amounted to 14.47 mEq O_2_/kg fat and 18.76 mEq O_2_/kg fat, respectively. The PV of pumpkin seed oil could be influenced by the variety and quality of the seeds and thermal treatment.

After two months of storage, only for HO stored at 4 °C, the PV increased approx. twice (12.34 ± 0.07 mEq O_2_/kg fat) and at 20 °C, almost fivefold (35.79 ± 1.52 mEq O_2_/kg fat). In the remaining samples, the increase in the tested parameter usually occurred only at room temperature. The PV permitted by the Codex Alimentarius [60] was exceeded in HO and all mixtures but only those stored at room temperature.

After four months of storage, the PV of the tested oils increased. A significant and largest increase (almost threefold) was observed at refrigerated temperature for HO (29.30 ± 0.20 mEq O_2_/kg fat). At room temperature, a large increase in PV (about twofold) occurred for HO and 3H:1P and 1H:3P mixtures. The PV for these samples was 71.23 ± 0.87, 66.57 ± 0.14, and 64.32 ± 0.06 mEq O_2_/kg fat. Interestingly, PV for the 1H:1P mixture was lower than that of the 3H:1P and 1H:3P mixtures, averaging 27.63 ± 0.14 mEq O_2_/kg fat. The permissible PV value was exceeded in HO stored at each temperature and in all mixtures stored at 20 °C.

Oils and their mixtures are generally less stable when stored at higher temperatures compared to lower temperatures. According to Van’t Hoff’s rule, when the temperature increases by 10 °C, the reaction rate increases by two to four times. The influence of oxygen on the oxidation rate depends, among other factors, on its temperature and concentration [73]. In the research conducted by Lo Turco et al. [59], the PV of hemp oil (filtered) stored for 12 weeks (at room temperature) from the beginning of storage (8.21 mEq O_2_/kg fat) increased to 13.38 mEq O_2_/kg of fat. In the mentioned studies, the oil was stored in transparent bottles, so the increase in PV was largely influenced by photosensitized oxidation.

Prescha et al. [66] also reported an increase in the PV of hemp oil after 6 months of storage at 20 °C from 3.23 ± 0.90 mEq O_2_/kg fat to 8.66 ± 6.39. In the study by Wroniak and Cenkier [67], the PV of oil from roasted pumpkin seeds after two months of storage at a refrigerated temperature increased from approx. 12 to 20 mEq O_2_/kg. Such high values could be due to the low quality of the raw material or the use of oil with a longer storage time after production. In the studies of Nosenko et al. [68], the PV of oil from unroasted pumpkin seeds increased from approx. 2 mEq O_2_/kg fat before the storage stage to ~10 after 98 days of storage. It is unknown under what conditions the storage tests were performed, but the PV changed gradually, similar to the tests in this study. Even though the oil was not produced from heat-treated seeds, the values obtained were lower than those analyzed. In the research of Petkova et al. [69], the longer the storage time (maximum 4 months) and temperature of pumpkin oil, the higher the PV—1.5–81.4 mEq O_2_/kg fat (25 °C) and 1.5–29 mEq O_2_/kg fat (10 °C). The low initial values were probably due to the good quality of the seeds, but the lack of thermal treatment of the seeds could cause quite high final results and their significant increase over time.

An interesting case turned out to be the 1H:1P mixture, in which the content of the hydroperoxides formed was lower after two months of storage at room temperature and after four months of storage at both refrigerated and room temperatures than in the mixture with a higher content of PO (1H:3P). No justification for this phenomenon has been found in the scientific literature. The probable cause could be antioxidants derived from hemp and pumpkin, which demonstrated a synergistic effect when combined in the appropriate proportion. Antioxidants originally found in oil are hydrogen donors, thanks to which they contribute to quenching radicals and interrupting the autoxidation chain reaction. They delay the auto-oxidation process (since the antioxidants themselves undergo the reaction, not the substrates) until they are used up [23]. The effectiveness of antioxidants depends, among others, on their chemical reactivity and interactions with food ingredients [74]. According to the literature, the primary antioxidants found in hemp oil include primarily phenolic compounds (188 mg/100 g of oil), γ-tocopherol (80–150 mg/100 g), phytosterols (3.9–6.7 mg/100 g), and β-carotene (3.36–5.34 mg/100 g) [9,61,75]. However, pumpkin seed oil contains antioxidants such as squalene (approx. 600/100 g of oil), δ-tocopherol (approx. 177 mg/100 g), β-sitosterol (84.40–116.33 mg/100 g), and phenolic compounds (mainly syringic acid—approx. 7.96 mg/100 g of oil and ferulic acid—approx. 4.99 mg/100 g of oil) [10,11,14,15,16,52]. Furthermore, the PO used in this study was roasted, which could have caused the formation of secondary antioxidants—Maillard reaction products. These include, among others, melanoidins that can delay or inhibit lipid oxidation reactions by scavenging free radicals, removing reactive oxygen species, and chelating transition metals [76]. Studies indicate that phytosterols accelerate the oxidation process independently, but with other compounds, they act synergistically to delay oxidative changes [23]. Tocopherols, acting synergistically with polyphenols, compete with unsaturated FAs for peroxide and alkoxy radicals, thereby reducing the degree of autoxidation. Squalene is also believed to be involved in scavenging peroxide radicals. Carotenoids primarily slow down the photosensitized oxidation process, so it can be assumed that their contribution to the capture of free radicals was small because the access to light was reduced as much as possible. However, they can still cooperate with chlorophyll pigments in quenching singlet oxygen (during photooxidation) [59]. The issue related to the characteristic properties of the 1H:1P mixture requires further research to determine the synergism of antioxidants and FAs present in it.

Statistical analysis showed the influence of the increase in temperature and extension of storage time on the increase in the content of hydroperoxides in the tested oils and their mixtures. Typically, the PV was lower in samples with higher PO content. The exception was the 1H:1P mixture, in which a lower PV was observed during storage than in the mixture with a higher content of PO.

### 3.4. Evaluation of Oil Oxidative Stability

The oxidative stability of fats is the resistance to oxidation during storage and processing, expressed as the time required to reach the oxidation critical point. The duration of the first stage of the autoxidation process (initiation) is a determinant of oxidative stability. The longer this time is, the better the stability of the oils [23]. Due to the relatively long oxidation time of oils at room temperature, accelerated oxidation tests are increasingly practiced, using methods in which the temperature increases or light exposure increases. One such method is pressure differential scanning calorimetry (PDSC) [77].

The average values of the PDSC oxidation time (τ_max_) of the tested oils and their mixtures are presented in Table 3. Before storing oils and their mixtures, the shortest oxidation induction time was characterized by HO (30.36 ± 1.20 min) and the longest by PO (111.38 ± 10.84 min). The statistical analysis showed that there were strong correlations between the oxidation induction time of individual samples and the FA-profile-positive for SFA (r = 0.86) and MUFA (r = 0.86) and negative for PUFA (r = −0.86). Therefore, one of the most important factors determining the relationship, according to which as the content of PO in the tested samples increased, their oxidative stability increased, was the composition of fatty acids. It is known that linoleic fatty acid oxidation is 10–40 times higher than that of oleic one, and the rate of linolenic oxidation is 2–4 times faster than that of linoleic [35]. PUFAs (including linoleic and linolenic FA) predominated in HO, and PO contained relatively many SFA and MUFA. As a result, HO underwent autoxidation faster [73]. Additionally, HO was probably pressed from poor-quality seeds, and PO was roasted, which could have resulted in the formation of secondary antioxidants (melanoidins), weakening the effect of free radicals and simultaneously enriching the antioxidant profile of the oil. There was also a strong negative correlation between individual samples’ PDSC oxidation time and AV (r = −0.87) and a moderate negative correlation between the oxidation time and the PV (r = −0.52).

Islam et al. [77] determined the oxidation time of HO (under similar conditions) in the 41.89–58.18 min range. The values were over 10 min higher than those obtained in this study. The HO used in the present paper was of poor quality, as evidenced by its acid and peroxide values. Hence, there is a large difference in its oxidative stability compared to the hemp oil in the studies of the scientists mentioned above. In the study by Bryś et al. [78], the PDSC oxidation time of HO was close to 29 min.

In the research conducted by Symoniuk et al. [79], the PDSC oxidation time of pumpkin seed oil (under similar conditions) was, on average, 71.05 min, which was over 30 min shorter than the oxidation induction time obtained for PO, which is the subject of the research in this study, which may be related to the lack of roasting of the seeds. The authors also indicated that the chlorophyll and carotenoid content significantly impacted the stability of the different cold-pressed oils.

After two months of storage, in the case of HO and 1H:1P and 1H:3P mixtures, the PDSC oxidation time was shortened only for samples stored at room temperature, while for the remaining samples, the oxidation time was shortened both after storage at refrigerated and room temperature. PO showed no statistically significant differences in oxidative stability depending on storage temperature (*p* > 0.05).

After four months of storage of HO at both temperatures and 3H:1P mixture at 20 °C, the oxidation time was shortened, while for PO, it was prolonged at both temperatures, reaching a value similar to the value from the period before its storage. In the case of 1H:1P and 1H:3P mixtures, oxidative stability did not change (*p* > 0.05).

A higher storage temperature accelerated the formation of primary oxidation products, resulting in poorer oxidative stability of oils and their mixtures [73]. Characteristic changes related to oxidative stability determined using PDSC after four months of storage, which in the case of some samples did not change or even increase, could be related to the relationship according to which the concentration of peroxides in oils increases until it reaches a maximum value and then gradually decreases as they decompose into secondary oxidation products [80]. The duration of this transformation varies depending on the type of oil. For example, in rapeseed oil and olive oil, secondary oxidation products are formed immediately after the formation of hydroperoxides, and in safflower and sunflower oil, only after the accumulation of a significant amount of primary oxidation products [58]. Research indicates that the thermal decomposition of oils occurs in several stages. Initially, polyene fatty acids and less stable oil components are decomposed and oxidized, followed by the loss of MUFAs and SFAs, and finally, condensation and pyrolysis products are oxidized. Thus, a lower content of PUFAs in the sample may delay its decomposition [73].

Storage time and temperature influenced the oxidative stability of oils and their mixtures to varying degrees. After two months of storage, the oxidation time was shortened for all samples. After four months, a shortening of the tested parameter was observed only in the case of HO and the 3H:1P mixture, while in the case of the remaining mixtures, the oxidation time did not change, and in the case of PO, it reached the value from the period before storage. In all samples except PO, a higher storage temperature worsened their oxidative stability.

It should be underlined that the stability of cold-pressed oil blends depends not only on the fatty acids composition but also on the content of antioxidants, primary and secondary oxidation products, metals, and other contaminants that might accelerate or inhibit oxidation [79]. Individual vegetable oils are a rich source of various substances with antioxidant activity. Blending different oils can cause the mixture of antioxidants from individual oils to exhibit synergistic activity at a specific concentration. The mechanism responsible for the synergistic antioxidant activity of the antioxidant mixture has not been explained so far, mainly due to the complex nature of the mixture [81]. According to the research of Shi et al. [82], the synergistic effect of the antioxidant depends on the type of antioxidant and its concentration. The authors indicated that some antioxidants (for example lycopene) interact synergistically with vitamin E but only at a specific concentration and ratio. The synergistic effect was not linearly dependent on the concentration of lycopene: the mixture containing its highest concentration did not exhibit the synergistic effect. Also, Ardabili et al. [83] indicated that the pumpkin oil levels higher than 5% in the mixture with canola oil mainly exerted the pro-oxidant effects, indicating the necessity of the stability investigations on its lower levels. There is therefore a great need to conduct more comprehensive studies aimed at the influence of the content of natural antioxidants found in a given oil mixture on the oxidative and hydrolytic stability of these mixtures. In our work, based on the results concerning oxidative stability, we put forward a hypothesis that requires verification that the composition of the mixture of different oils, and thus the composition of antioxidants found in a given mixture influences the oxidative stability of these mixtures.

## 4. Conclusions

Blending edible oils may lead to a mixture with improved nutritional and functional properties. However, with the development of the nutraceutical and functional foods market, more attention is now devoted to non-traditional vegetable oils and their blends. This study examined hemp and pumpkin seed oils and their blends during storage in typical household conditions.

The results showed that the time and temperature of the storage of oils and their mixtures did not affect the fatty acid composition of the tested samples. The greater the addition of pumpkin seed oil to the mixture, the lower the content of PUFA and the greater the content of SFA and MUFA. The obtained mixtures contained mainly unsaturated FAs in the central TAG position and SFA in the external positions. From the nutritional point of view, such an arrangement is not advantageous because, during digestion, insoluble salts may be formed by combining saturated long-chain fatty acids with calcium or magnesiumions.

The extension of the storage time and temperature of the tested oils and their mixtures resulted in an increase in the content of FFAs and the content of primary oxidation products in the samples. The exception was PO, for which statistical analysis did not show a significant effect of temperature on the AV.

The 1H:1P (*v*:*v*) mixture was characterized by a satisfactory AV and PV during storage, a desirable FA profile, and a relatively good PDSC oxidation time. The distribution of fatty acids in TAG molecules in this mixture variant was not favorable from the point of view of digestibility. However, due to the excellent nutritional value, the balanced ratio of n-3 to n-6 acids, and good hydrolytic and oxidative stability, a mixture variant with such a proportion of HO to PO seems to be the most beneficial. The specific properties of the 1H:1P mixture may result from the synergistic effect of bioactive compounds from both oils mixed in equal amounts. Nevertheless, this issue requires further research.

The presented results may be a kind of guidance on how to select the appropriate proportions of hemp and pumpkin seed oils to obtain blends of favorable stability and nutritional quality. The limitations of this paper are related to the study of oils from only one producer and only one plant variety. It should also be realized that, apart from the variety, the chemical composition and quality of the oil derived from the seeds depends significantly on the quality of the seeds influenced by, among others, climate, environmental factors, growing and harvesting conditions of the plants, as well as the degree of their maturity, but also on the processing and storage conditions of oil. Nonetheless, it has been proven that cold-pressed hemp oil can be used as an additive to other oils to improve the ratio of n-6 to n-3, but its high content has a negative impact on oxidative stability; therefore, it should be blended with an oil of high stability, such as pumpkin oil.

## Figures and Tables

**Table 1:** Health indices of tested oils and their blends immediately after opening and after two and four months of storage at 4 °C and 20 °C.

Sample	Storage [Month]	Temperature [°C]	AI	TI	HPI	h/H
H	0	-	0.07	0.11	15.35	15.55
	2	4	0.08	0.12	12.47	12.71
		20	0.07	0.11	14.85	15.03
	4	4	0.07	0.11	13.92	14.08
		20	0.07	0.11	13.92	14.06
3H:1P	0	-	0.09	0.16	11.46	11.65
	2	4	0.09	0.16	11.57	11.72
		20	0.09	0.16	11.44	11.59
	4	4	0.09	0.16	10.75	10.97
		20	0.10	0.17	10.03	10.25
1H:1P	0	-	0.11	0.22	9.29	9.47
	2	4	0.12	0.24	8.23	8.40
		20	0.11	0.22	9.29	9.49
	4	4	0.12	0.24	8.23	8.40
		20	0.13	0.24	7.87	8.03
1H:3P	0	-	0.13	0.32	7.44	7.62
	2	4	0.13	0.32	7.59	7.77
		20	0.14	0.32	7.39	7.55
	4	4	0.14	0.33	7.25	7.43
		20	0.15	0.34	6.89	7.05
P	0	-	0.15	0.48	6.47	6.65
	2	4	0.16	0.49	6.35	6.53
		20	0.16	0.48	6.35	6.53
	4	4	0.17	0.51	5.89	6.04
		20	0.17	0.50	5.89	6.05

H—hemp oil, 3H:1P—3:1 (*v*/*v*) hemp oil to pumpkin seed oil mixture, 1H:1P—1:1 (*v*/*v*) hemp oil to pumpkin seed oil mixture, 1H:3P—1:3 (*v*/*v*) hemp oil to pumpkin seed oil mixture, P—pumpkin seed oil. AI—atherogenic index; TI—thrombogenic index; HPI—health promoting index; h/H—hypocholesterolaemic/hypercholesterolaemic ratio.

**Table 2:** Average acid (AV) and peroxide values (PV) of tested oils and their mixtures immediately after opening and after two and four months of storage at 4 °C and 20 °C.

Sample	Storage [Month]	Temperature [°C]	AV [mg KOH/g Oil]	PV [meq O_2_/kg Oil]
H	0	-	4.25 ^e^ ± 0.01	7.24 ^e^ ± 0.14
	2	4	4.84 ^d^ ± 0.01	12.34 ^d^ ± 0.07
		20	4.97 ^c^ ± 0.02	35.79 ^b^ ± 1.52
	4	4	5.54 ^b^ ± 0.01	29.30 ^c^ ± 0.20
		20	5.81 ^a^ ± 0.01	71.23 ^a^ ± 0.87
3H:1P	0	-	3.34 ^e^ ± 0.01	6.17 ^d^ ± 0.05
	2	4	3.81 ^d^ ± 0.01	7.34 ^d^ ± 0.60
		20	3.95 ^c^ ± 0.05	28.43 ^b^ ± 1.23
	4	4	4.36 ^b^ ± 0.01	13.13 ^c^ ± 0.15
		20	4.62 ^a^ ± 0.01	66.57 ^a^ ± 0.14
1H:1P	0	-	2.47 ^e^ ± 0.01	5.63 ^d^ ± 0.26
	2	4	2.80 ^d^ ± 0.01	6.58 ^cd^ ± 0.44
		20	2.87 ^c^ ± 0.01	16.63 ^b^ ± 1.34
	4	4	3.20 ^b^ ± 0.01	8.83 ^c^ ± 0.49
		20	3.42 ^a^ ± 0.01	27.63 ^a^ ± 0.14
1H:3P	0	-	1.51 ^e^ ± 0.01	5.24 ^d^ ± 0.07
	2	4	1.76 ^d^ ± 0.01	5.54 ^d^ ± 0.59
		20	1.79 ^c^ ± 0.01	33.48 ^b^ ± 0.41
	4	4	1.98 ^b^ ± 0.01	10.02 ^c^ ± 1.02
		20	2.06 ^a^ ± 0.01	64.32 ^a^ ± 0.06
P	0	-	0.58 ^c^ ± 0.01	4.81 ^d^ ± 0.12
	2	4	0.63 ^b^ ± 0.01	5.18 ^d^ ± 0.11
		20	0.61 ^b^ ± 0.01	8.44 ^c^ ± 0.54
	4	4	0.76 ^a^ ± 0.01	10.32 ^b^ ± 0.64
		20	0.78 ^a^ ± 0.01	12.66 ^a^ ± 0.50

H—hemp oil, 3H:1P—3:1 (*v*/*v*) hemp oil to pumpkin seed oil mixture, 1H:1P—1:1 (*v*/*v*) hemp oil to pumpkin seed oil mixture, 1H:3P—1:3 (*v*/*v*) hemp oil to pumpkin seed oil mixture, P—pumpkin seed oil. The different lowercase letters indicate significantly different values (*p* ≤ 0.05). Data are presented as mean values followed by standard deviation (±SD).

**Table 3:** PDSC oxidation time (τ_max_) of tested oils and their mixtures at 120 °C immediately after opening and after two and four months of storage at 4 °C and 20 °C.

Sample	Storage [Month]	Temperature [°C]	τ_max_ [Min]
H	0	-	30.36 ^a^ ± 1.20
	2	4	28.41 ^a^ ± 1.07
		20	21.82 ^b^ ± 0.57
	4	4	23.07 ^b^ ± 0.08
		20	15.21 ^c^ ± 0.17
3H:1P	0	-	38.60 ^a^ ± 1.07
	2	4	34.01 ^b^ ± 0.99
		20	26.60 ^c^ ± 0.25
	4	4	35.33 ^ab^ ± 1.36
		20	20.32 ^d^ ± 1.33
1H:1P	0	-	48.86 ^a^ ± 1.56
	2	4	43.56 ^ab^ ± 0.35
		20	38.93 ^b^ ± 0.61
	4	4	47.50 ^a^ ± 2.27
		20	39.33 ^b^ ± 2.16
1H:3P	0	-	61.18 ^a^ ± 1.75
	2	4	53.26 ^a^ ± 1.24
		20	39.26 ^b^ ± 4.44
	4	4	66.26 ^a^ ± 5.69
		20	38.27 ^b^ ± 0.59
P	0	-	111.38 ^a^ ± 10.84
	2	4	72.46 ^b^ ± 1.78
		20	65.46 ^b^ ± 1.08
	4	4	97.86 ^a^ ± 3.53
		20	96.04 ^a^ ± 3.06

H—hemp oil, 3H:1P—3:1 (*v*/*v*) hemp oil to pumpkin seed oil mixture, 1H:1P—1:1 (*v*/*v*) hemp oil to pumpkin seed oil mixture, 1H:3P—1:3 (*v*/*v*) hemp oil to pumpkin seed oil mixture, P—pumpkin seed oil. Determined data are presented as mean values followed by standard deviation (±SD). The different lower-case letters indicate significantly different values (*p* ≤ 0.05).

## Data Availability

The original contributions presented in the study are included in the article/Supplementary Materials, further inquiries can be directed to the corresponding author.

## Supplementary Materials

The following supporting information can be downloaded at: https://www.mdpi.com/article/10.3390/foods13233813/s1, Table S1. Fatty acid composition [%] of tested oils and their blends immediately after opening and after two and four months of storage at 4 °C; Table S2. Fatty acid composition [%] of tested oils and their blends after two and four months of storage at 20 °C.
