# Supplementary material for: Assessment of the Stability and Nutritional Quality of Hemp Oil and Pumpkin Seed Oil Blends
Source: Foods. 2024 Nov 26;13(23):3813. doi: 10.3390/foods13233813 (PMC11639808; doi:10.3390/foods13233813)
Supplement: Supplementary file 1 [file foods-13-03813-s001.zip › foods-3269260-supplementary.pdf]

Table S1. Fatty acid composition [%] of tested oils and their blends immediately after opening and after two and four months of storage at 4°C.

	Composition after opening [%]					Composition after 2 months of storage [%]					Composition after 4 months of storage [%]				
**Fatty acid**	**H**	**3H:1P**	**1H:1P**	**1H:3P**	**P**	**H**	**3H:1P**	**1H:1P**	**1H:3P**	**P**	**H**	**3H:1P**	**1H:1P**	**1H:3P**	**P**
**C14:0**	0.04±0.01	0.06±0.01	0.08±0.01	0.11±0.01	0.13±0.01	0.06±0.02	0.05±0.01	0.09±0.01	0.10±0.01	0.13±0.01	0.04±0.01	0.07±0.01	0.09±0.01	0.11±0.01	0.13±0.01
**C16:0**	5.58±0.96	7.26±0.01	8.72±0.01	10.54±0.43	11.81±0.31	6.79±1.41	7.25±0.01	9.70±0.07	10.40±0.33	12.04±0.19	6.16±0.07	7.71±0.10	9.78±0.70	10.82±0.12	12.90±0.28
**C16:1**	0.11±0.04	0.12±0.01	0.13±0.01	0.14±0.01	0.14±0.01	0.10±0.01	0.11±0.01	0.14±0.01	0.14±0.01	0.14±0.01	0.11±0.01	0.13±0.01	0.14±0.01	0.14±0.01	0.15±0.01
**C17:0**	0.08±0.01	0.07±0.01	0.07±0.01	0.06±0.01	0.06±0.01	0.08±0.03	0.06±0.01	0.06±0.01	0.06±0.01	0.05±0.01	0.07±0.01	0.06±0.01	0.06±0.01	0.05±0.01	0.06±0.01
**C17:1**	0.03±0.01	0.03±0.01	0.03±0.01	0.02±0.01	0.02±0.01	0.05±0.01	0.03±0.01	0.03±0.01	0.02±0.01	0.02±0.01	0.04±0.01	0.02±0.02	0.03±0.01	0.03±0.01	0.02±0.01
**C18:0**	3.70±0.11	4.65±0.01	5.59±0.01	6.45±0.08	7.49±0.09	3.39±0.24	4.61±0.01	5.30±0.19	6.41±0.03	7.38±0.03	3.60±0.06	4.49±0.02	5.30±0.20	6.30±0.01	7.28±0.05
**C18:1 n-9**	14.10±0.18	18.05±0.01	21.93±0.05	25.82±0.12	29.95±0.09	13.68±0.30	18.10±0.02	21.71±0.24	25.82±0.07	30.00±0.08	14.08±0.08	17.93±0.03	21.72±0.22	25.71±0.01	29.79±0.06
**C18:2 n-6**	52.87±0.11	51.44±0.01	50.27±0.06	49.13±0.25	48.46±0.20	52.93±0.45	51.82±0.08	50.36±0.07	49.63±0.22	48.72±0.18	53.11±0.15	51.66±0.02	50.36±0.07	49.32±0.09	48.03±0.06
**C18:3 n-6**	2.35±0.08	1.87±0.01	1.29±0.01	0.67±0.01	-	2.39±0.04	1.88±0.01	1.31±0.04	0.68±0.01	-	2.38±0.03	1.95±0.04	1.31±0.04	0.68±0.01	-
**C18:3 n-3**	17.36±0.30	13.34±0.01	9.21±0.02	4.90±0.01	0.22±0.01	17.55±0.14	13.29±0.02	9.07±0.04	4.90±0.01	0.22±0.01	17.23±0.06	13.32±0.01	9.07±0.04	4.87±0.01	0.21±0.01
**C20:0**	1.23±0.24	1.04±0.01	0.90±0.04	0.74±0.01	0.63±0.01	0.89±0.23	0.92±0.01	0.69±0.10	0.66±0.01	0.56±0.01	1.00±0.02	0.84±0.03	0.69±0.11	0.63±0.01	0.48±0.03
**C20:1 n-9**	0.57±0.09	0.44±0.01	0.34±0.01	0.23±0.01	0.12±0.01	0.41±0.12	0.49±0.01	0.27±0.04	0.25±0.01	0.06±0.06	0.48±0.01	0.38±0.01	0.27±0.04	0.20±0.01	0.11±0.02

**C20:4 n-6**	0.60±0.18	0.43±0.01	0.36±0.02	0.27±0.01	0.15±0.01	0.45±0.05	0.33±0.04	0.22±0.10	0.20±0.01	0.15±0.01	0.43±0.05	0.27±0.05	0.22±0.01	0.22±0.01	0.15±0.01
**C22:2**	0.13±0.07	0.19±0.01	0.34±0.01	0.42±0.03	0.57±0.01	0.07±0.01	0.18±0.01	0.27±0.08	0.32±0.01	0.42±0.02	0.09±0.01	0.20±0.01	0.26±0.08	0.44±0.02	0.48±0.04
**C24:0**	0.27±0.08	0.19±0.01	0.18±0.01	0.16±0.02	0.15±0.01	0.15±0.01	0.14±0.01	0.14±0.04	0.12±0.01	0.07±0.01	0.18±0.01	0.15±0.04	0.14±0.04	0.15±0.02	0.12±0.01
**C24:1**	0.03±0.01	0.05±0.01	0.07±0.01	0.10±0.02	0.11±0.04	0.03±0.01	0.05±0.01	0.08±0.01	0.06±0.01	0.06±0.06	0.05±0.01	0.07±0.01	0.08±0.01	0.10±0.01	0.10±0.01
**other**	0.99±0.04	0.81±0.01	0.55±0.01	0.29±0.01	0.02±0.01	1.03±0.01	0.77±0.01	0.53±0.02	0.27±0.01	0.02±0.01	0.99±0.01	0.80±0.01	0.53±0.02	0.29±0.01	0.02±0.01

H – hemp oil, 3H:1P – 3:1 (v/v) hemp oil to pumpkin seed oil mixture, 1H:1P – 1:1 (v/v) hemp oil to pumpkin seed oil mixture, 1H:3P – 1:3 (v/v) hemp oil to pumpkin seed oil mixture, P – pumpkin seed oil
Data are presented as mean values followed by standard deviation (±SD)

Table S2. Fatty acid composition [%] of tested oils and their blends after two and four months of storage at 20°C.

	**Composition after 2 months of storage [%]**					**Composition after 4 months of storage [%]**				
**Fatty acid**	**H**	**3H:1P**	**1H:1P**	**1H:3P**	**P**	**H**	**3H:1P**	**1H:1P**	**1H:3P**	**P**
**C14:0**	0.04±0.01	0.05±0.01	0.08±0.01	0.10±0.01	0.13±0.01	0.04±0.01	0.08±0.01	0.11±0.01	0.11±0.01	0.14±0.01
**C16:0**	5.78±0.03	7.34±0.04	8.76±0.04	10.68±0.15	12.05±0.16	6.15±0.01	8.20±0.33	10.19±0.40	11.35±0.04	12.89±0.40
**C16:1**	0.10±0.01	0.12±0.01	0.11±0.01	0.11±0.01	0.15±0.01	0.12±0.01	0.13±0.01	0.15±0.01	0.15±0.01	0.16±0.01
**C17:0**	0.07±0.01	0.06±0.01	0.06±0.01	0.06±0.01	0.06±0.01	0.08±0.01	0.07±0.01	0.06±0.01	0.07±0.01	0.07±0.01
**C17:1**	0.04±0.01	0.03±0.01	0.03±0.01	0.01±0.01	0.03±0.01	0.04±0.01	0.04±0.01	0.03±0.01	0.02±0.01	0.02±0.01

**C18:0**	3.67±0.01	4.57±0.01	5.37±0.01	6.34±0.01	7.35±0.01	3.70±0.01	4.40±0.04	5.24±0.19	6.21±0.01	7.09±0.10
**C18:1 n-9**	14.09±0.01	18.02±0.01	21.68±0.02	25.72±0.14	29.97±0.05	14.23±0.01	17.75±0.02	21.58±0.14	25.64±0.03	29.58±0.13
**C18:2 n-6**	52.96±0.01	51.88±0.01	50.90±0.04	49.98±0.08	48.79±0.18	52.70±0.01	51.47±0.02	50.18±0.21	49.30±0.04	48.50±0.15
**C18:3 n-6**	2.53±0.01	1.91±0.01	1.33±0.02	0.68±0.01	-	2.53±0.01	1.99±0.01	1.31±0.03	0.68±0.01	-
**C18:3 n-3**	17.41±0.01	13.36±0.01	9.51±0.01	4.92±0.01	0.22±0.01	17.06±0.01	13.29±0.01	8.93±0.04	4.79±0.01	0.22±0.01
**C20:0**	1.11±0.01	0.82±0.03	0.72±0.06	0.61±0.01	0.51±0.03	1.08±0.01	0.79±0.05	0.68±0.08	0.58±0.01	0.48±0.02
**C20:1 n-9**	0.56±0.02	0.49±0.01	0.30±0.07	0.21±0.04	0.10±0.01	0.56±0.01	0.35±0.01	0.25±0.02	0.17±0.01	0.10±0.01
**C20:4 n-6**	0.44±0.01	0.31±0.01	0.25±0.01	0.19±0.01	0.14±0.01	0.50±0.01	0.19±0.23	0.26±0.03	0.19±0.01	0.14±0.01
**C22:2**	0.04±0.01	0.16±0.01	0.20±0.01	0.24±0.01	0.38±0.01	0.04±0.01	0.21±0.01	0.29±0.04	0.25±0.01	0.41±0.01
**C24:0**	0.16±0.01	0.13±0.01	0.12±0.01	0.10±0.01	0.10±0.04	0.16±0.01	0.18±0.01	0.16±0.01	0.14±0.01	0.10±0.01
**C24:1**	0.04±0.01	0.04±0.01	0.05±0.01	0.06±0.01	0.05±0.02	0.05±0.01	0.07±0.01	0.07±0.01	0.09±0.01	0.12±0.01
**other**	1.00±0.01	0.78±0.01	0.56±0.01	0.30±0.01	0.02±0.01	1.01±0.01	0.82±0.01	0.53±0.01	0.28±0.01	0.02±0.01

H – hemp oil, 3H:1P – 3:1 (v/v) hemp oil to pumpkin seed oil mixture, 1H:1P – 1:1 (v/v) hemp oil to pumpkin seed oil mixture, 1H:3P – 1:3 (v/v) hemp oil to pumpkin seed oil mixture, P – pumpkin seed oil

Data are presented as mean values followed by standard deviation (±SD)
